# Computational Analysis of siRNA Recognition by the Ago2 PAZ Domain and Identification of the Determinants of RNA-Induced Gene Silencing

**DOI:** 10.1371/journal.pone.0057140

**Published:** 2013-02-18

**Authors:** Mahmoud Kandeel, Yukio Kitade

**Affiliations:** 1 United Graduate School of Drug Discovery and Medical Information Sciences, Gifu University, Gifu, Japan; 2 Department of Biomolecular Science, Faculty of Engineering, Gifu University, Gifu, Japan; 3 Department of Pharmacology, Faculty of Veterinary Medicine, Kafrelshikh University, Kafrelshikh, Egypt; The John Curtin School of Medical Research, Australia

## Abstract

RNA interference (RNAi) is a highly specialized process of protein-siRNA interaction that results in the regulation of gene expression and cleavage of target mRNA. The PAZ domain of the Argonaute proteins binds to the 3' end of siRNA, and during RNAi the attaching end of the siRNA switches between binding and release from its binding pocket. This biphasic interaction of the 3' end of siRNA with the PAZ domain is essential for RNAi activity; however, it remains unclear whether stronger or weaker binding with PAZ domain will facilitate or hinder the overall RNAi process. Here we report the correlation between the binding of modified siRNA 3' overhang analogues and their *in vivo* RNAi efficacy. We found that higher RNAi efficacy was associated with the parameters of lower Ki value, lower total intermolecular energy, lower free energy, higher hydrogen bonding, smaller total surface of interaction and fewer van der Waals interactions. Electrostatic interaction was a minor contributor to compounds recognition, underscoring the presence of phosphate groups in the modified analogues. Thus, compounds with lower binding affinity are associated with better gene silencing. Lower binding strength along with the smaller interaction surface, higher hydrogen bonding and fewer van der Waals interactions were among the markers for favorable RNAi activity. Within the measured parameters, the interaction surface, van der Waals interactions and inhibition constant showed a statistically significant correlation with measured RNAi efficacy. The considerations provided in this report will be helpful in the design of new compounds with better gene silencing ability.

## Introduction

RNA interference (RNAi) is a cellular process triggered by double stranded RNA(dsRNA) and regulates the gene expression of target mRNA [1], [2]. The major players in this process are the Dicer and Argonaute proteins (Agos). Dicer is involved in cleavage of microRNA (miRNA) into small interfering RNA (siRNA), whereas Agos are the catalytic components of RNA-induced silencing complex (RISC) which bind to siRNAs and cleave mRNA targets [3], [4]. The RNAs class that binds with Ago protein, the siRNA, is characterized by the presence of two single nucleotides at their 3' overhangs. RNAi technology is considered a useful tool for controlling cancer and virus infection and other applications based on controlling of the expression of a molecular target [5]–[9]. Furthermore, modified 3' overhang analogues were an interesting target for development of potent RNAi efficacy [10]–[12].

The functional domains of Ago proteins involved in gene silencing process includes two binding domains (MID and PAZ domains) and one catalytic domain for cleavage of mRNA (PIWI domain). MID domain is involved in binding the 5' phosphate of siRNA [13], while PAZ domain is important for binding the 3' end of siRNA [14], [15]. The binding properties of MID domain are less dynamically variable than PAZ domain, thus underscoring its vulnerability for siRNA modifications [16], [17]. The events occurring at the binding of 3' nucleotide of siRNA with PAZ involve a series of interesting molecular dynamics during siRNA-Agos binding and cleavage mechanisms.

During the RNAse activity of RISC-RNA complex, the 3' end of siRNA toggles between binding and release from the binding cavity of Paz domain. The former changes were found to be essential for proper functioning of mRNA cleavage. The siRNA-mediated target cleavage cycle involves four proposed events [18]. The cycle starts by binding of siRNA with Ago which involves anchoring of 3' end the by PAZ domain followed by release of the passenger strand [19]. The second step involves base pairing with target mRNA. To maximize base pairing alignments, the base pairing starts at 5' end and flares up to the 3' end causing a rotational transition of the PAZ domain away from the N domain, thereby releasing the 3′ end of the guide strand from the PAZ. The third step involves cleavage of target RNA. Finally, as the cleaved products release from Ago2, PAZ domain returns back to its 3' end binding alignment. Interestingly, it was reported that Ago2 undergoes significant conformational changes upon binding with target RNAs of different lengths. In comparison with 12-nucleotide target RNA, the binding of a 15-nucleotide target RNA is accompanied by pivotal rotation of PAZ domain [20]. Recently, it was reported that PAZ domain is essential in RNAi process. PAZ-disrupted Ago mutants were unable to unwind or eject the passenger strand of miRNA-like mismatch-containing duplexes [21].

During RNAi, events occurring at the 3′ end of siRNA involve binding of the PAZ domain with the nucleotide or compound at this position, followed by release and rebinding in a cyclic manner. In this context, several modifications of the nucleotides at the 3' end have been thoroughly investigated [10], [22]–[25]. However, it is not well understood whether compounds with stronger or weaker binding with PAZ domain could enhance or hinder the whole RNAi process. The main goal of this study was to explore the impact of weaker or stronger binding of siRNA on overall RNAi effects. It is proposed that stronger binding with the PAZ domain might interfere with the previously mentioned siRNA binding-release cycle, thereby affecting the whole RNAi process. For this purpose, we analyzed the experimentally determined *in vivo* activities of siRNAs produced previously by our lab and then correlated these results with computational and modeling tools. In this study, several questions have to be addressed regarding to, what are the forces governing 3' recognition by PAZ domain?, what is the relation between *in vivo* efficacy of modified siRNAs and the binding affinity of 3' overhangs?, the correlation between the size of modified 3' overhangs or the total interaction surface with PAZ domain and RNAi, and finally, what is the relation between strong or weak binding with PAZ domain and RNAi?.

## Methods

### Molecular docking studies

#### Preparation of compounds

**Figure 1:**
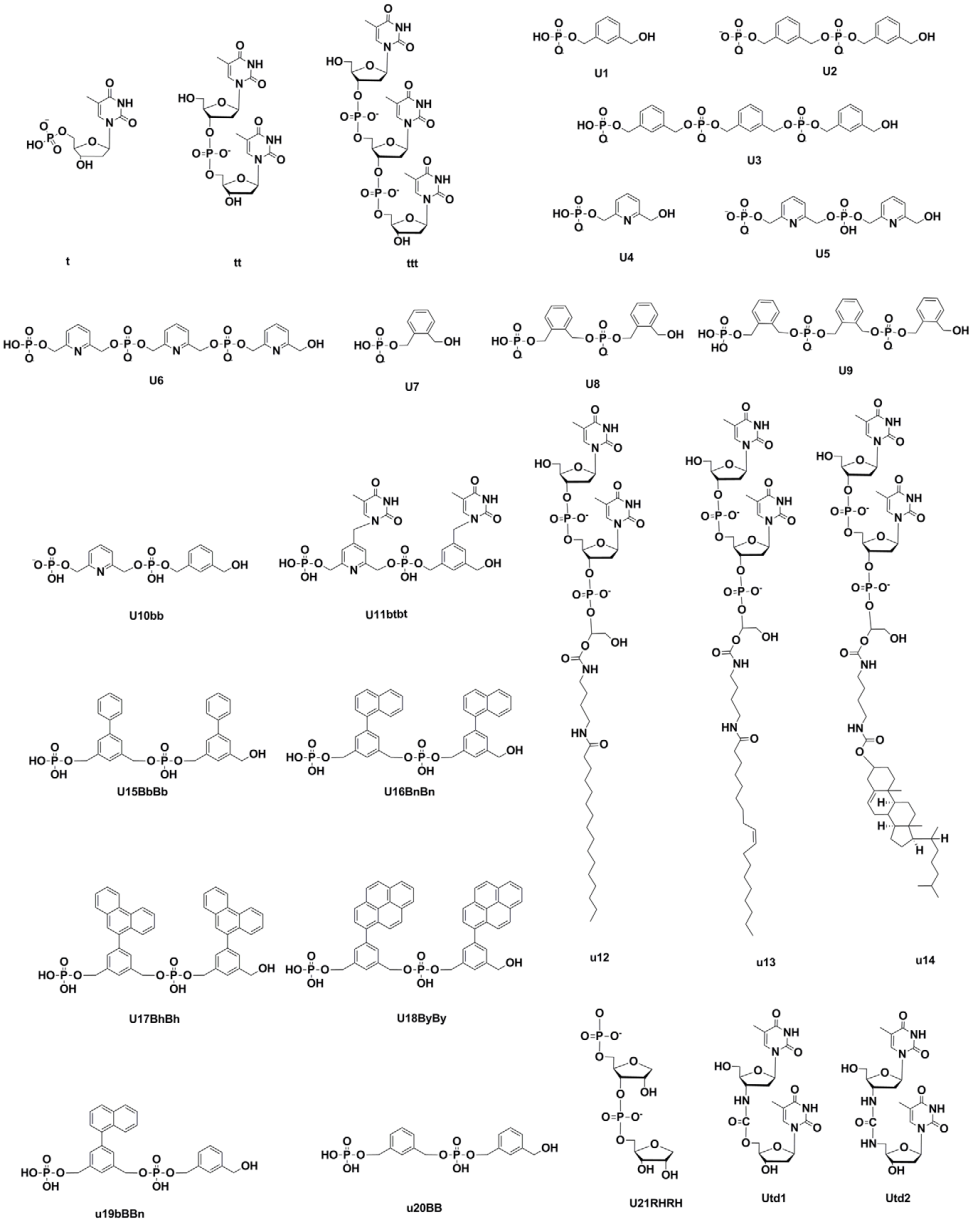
Structure of modified nucleotides or nucleotides analogues used in the docking studies. The figure is generated by ChemBioDraw ultra 12.0 (CambridgeSoft, USA).

Several siRNA 3' overhang modifications were developed in our lab [22], [26]–[32]. The structure of these compounds (as shown in Fig. 1) together with their *in vivo* efficacy were retrieved and subjected to further investigations including docking studies and computational tools. Compounds conformation and orientation relative to the binding site was computed by using a generic evolutionary method provided by iGEMDOC [33], [34]. Cleaning and optimization of compounds conformation was carried out by ChemSketch 12.01 software (ACDlabs, Canada). Hydrogens were removed and compounds saved as Mol files after file format conversion tools available with Openbabel software version 3.2.1.

#### Preparation of protein

The crystal structure of drosophila Ago2 was used for docking studies (PDB ID 3MJ0). The structure is containing one chain and the protein is bound with siRNA. The binding site is defined from the bound ligand and the binding site radius was set to 10Å.

*Docking*: Docking studies were performed by using iGEMDOCK as well as by using the automated functions available at the docking server (http://www.dockingserver.com/). The results of docking runs are given in Tables 1 and 2. In order to get accurate docking, stable (slow) docking was used as a default setting. Blind docking runs and repeats of runs with the same compounds were carried out to avoid false positive or false negative results.

**Table 1:** The docking results by using iGEMDOCK.

#Ligand	Total Energy	VDW	HBond	Elec	AverConPair	RL/FL
cav3MJ0_OMU-t-5.pdb	−104.318	−73.4979	−27.2992	−3.5205	23.1905	0.48
cav3MJ0_OMU-tt-1.pdb	−132.792	−92.246	−38.6308	−1.9147	18.7838	0.29
cav3MJ0_OMU-ttt-0.pdb	−161.759	−114.306	−44.7093	−2.7443	14.9016	0.28
cav3MJ0_OMU-u1-2.pdb	−81.8359	−59.2329	−19.3551	−3.2479	26.9286	0.48
cav3MJ0_OMU-u2-5.pdb	−122.043	−90.4323	−27.9656	−3.6454	21.5926	0.37
cav3MJ0_OMU-u3-1.pdb	−121.332	−78.1598	−38.5098	−4.6619	18.375	0.4
cav3MJ0_OMU-u4-9.pdb	−73.5425	−50.9181	−22.6244	0	22.5714	0.42
cav3MJ0_OMU-u5-3.pdb	−124.79	−78.7642	−39.3695	−6.6563	20.6296	0.32
cav3MJ0_OMU-u6-5.pdb	−131.49	−89.5491	−37.7314	−4.2097	17.075	0.34
cav3MJ0_OMU-u7-6.pdb	−82.3028	−47.6383	−32.569	−2.0955	24.8571	0.3
cav3MJ0_OMU-u8-4.pdb	−111.931	−66.4112	−40.0519	−5.4676	20.2963	0.32
cav3MJ0_OMU-u9-1.pdb	−136.815	−103.677	−26.0044	−7.1335	19.275	0.36
cav3MJ0_OMU-u10-3.pdb	−122.538	−74.458	−42.047	−6.0326	20.8519	0.2
cav3MJ0_OMU-u11btbt-7.pdb	−148.725	−114.562	−31.4952	−2.668	18.0638	0.37
cav3MJ0_OMU-u12-2.pdb	−132.798	−90.1461	−38.9404	−3.7118	15.1	0.6
cav3MJ0_OMU-u13-2.pdb	−144.691	−112.321	−31.9466	−0.4229	11.4444	0.65
cav3MJ0_OMU-u14-8.pdb	−142.09	−109.916	−33.939	1.76489	11.1325	1.1
cav3MJ0_OMU-u15bbbb-8.pdb	−134.383	−106.677	−23.3255	−4.3808	17.6154	0.58
cav3MJ0_OMU-u16-9.pdb	−155.5	−128.061	−24.3061	−3.1337	17.9149	0.55
cav3MJ0_OMU-u17bnbn-1.pdb	−147.465	−116.081	−24.1495	−7.235	19.0426	0.7
cav3MJ0_OMU-u18byby-9.pdb	−149.812	−127.371	−23.7485	1.30793	15.322	0.78
cav3MJ0_OMU-u19bbn-2.pdb	−130.645	−104.098	−23.4739	−3.0728	19.5946	1.05
cav3MJ0_OMU-u20bb-8.pdb	−118.559	−88.7504	−27.6149	−2.194	22.6296	0.74
cav3MJ0_OMU-u21rhrh-5.pdb	−119.638	−66.7059	−46.1571	−6.7748	20.28	0.19
cav3MJ0_OMU-utd1-8.pdb	−131.388	−96.4664	−34.922	0	19.3611	0.25
cav3MJ0_OMU-utd2-8.pdb	−125.788	−97.4032	−28.3847	0	19.2222	0.28

The ligands are ordered as shown in Fig. 1). The output data included total energy (Kcal/mol), van der Waals interactions (VDW, Kcal/mol), Hydrogen bonding (HBond, Kcal/mol), electrostatic interactions (elec, Kcal/mol) and average conpair. RL/FL indicates *Renilla* luciferace expression normalized by firefly luciferase data.

**Table 2:** The docking results by using the docking server.

Compound	Estimated free energy	Inhibition constant Ki	vDw + Hbond + desolvation energy	Electrostatic energy	Total intermolecular energy	Interaction surface	RL/FL
t	−6.9	8.82	−7.07	−0.08	−7.15	572.46	0.48
tt	−5.37	115.63	−6.6	−0.66	−7.27	732.72	0.29
ttt	−2.39	17.56	−4.15	−0.47	−4.62	727.216	0.28
U1	−5.24	143.04	−6.62	−0.08	−6.7	503.7	0.48
U2	−5.77	58.9	−8.26	−0.65	−8.91	747.37	0.37
U3	−4.63	402.26	−6.76	−1.35	−8.11	748.43	0.4
U4	−4.97	228.99	−5.55	−0.12	−5.67	466.14	0.42
U5	−5.9	47.67	−7.35	−1	−8.35	688	0.32
U6	−4.54	466.86	−7.25	−1.41	−8.66	899	0.34
U7	−5.25	140.99	−5.75	−0.04	−5.79	484	0.3
U8	−5.88	49.19	−7.16	−0.93	−8.09	667	0.32
U9	−4.6	424.55	−7.71	−1.38	−9.1	820	0.36
U10bbn	−5.63	74.8	−7.46	−0.83	−8.29	688	0.2
U11btbt	−6.03	38.27	−8.66	−0.79	−9.45	901.26	0.37
U12	174.9	nd	79.39	−1.94	77.46	1188	0.6
U13	15.67	nd	−4.8	−1.23	−6.03	995.45	0.65
U14	57.72	nd	9.17	−0.32	8.85	1117	1.1
U15BbBb	−5.87	49.61	−8.26	−0.75	−9.01	744	0.58
U16BnBn	−7.26	4.8	−7.9	−0.53	−8.43	901.48	0.7
U17BhBh	−6.68	12.75	−8.65	−0.62	−9.26	945.85	0.78
U18ByBy	−7.53	3	−10.06	−0.45	−10.5	808.5	1.05
u19bBBn	−5.97	42.37	−7.8	−0.66	−8.45	835.7	0.74
u20BB	−5.88	48.57	−7.89	−0.68	−8.57	719	0.26
Utd1	−5.87	57.82	−6.71	−0.18	−6.89	780.3	0.3
Utd2	−6.36	21.8	−6.4	−0.09	−6.49	664	0.28
U21RHRH	−5.67	60.14	−6.13	−0.9	−7.03	616.33	0.19

The ligands are ordered as shown in Fig. 1). The output data included free energy (Kcal/mol), inhibition constant (µM),van der Waals interactions, Hydrogen bonding and desolvation energy (Kcal/mol), electrostatic energy (Kcal/mol) and interaction surface. RL/FL indicates *Renilla* luciferace expression normalized by firefly luciferase data.

In iGEMDOCK, the parameters of docking run were set as population size (N = 300), generations (80), number of solutions (10). The best pose was selected based on the best conformation that allows the lowest free energy of binding.

The docking server [35] is based on MMFF94 force field for energy minimization of ligand molecules. Gasteiger partial charges were added to the ligand atoms. Non-polar hydrogen atoms were merged, and rotatable bonds were defined. Essential hydrogen atoms, Kollman united atom type charges, and solvation parameters were added with the aid of AutoDock tools. Affinity (grid) maps of 20×20×20 Å grid points and 0.375 Å spacing were generated using the Autogrid program. AutoDock parameter set- and distance-dependent dielectric functions were used in the calculation of the van der Waals and the electrostatic terms, respectively. Docking simulations were performed using the Lamarckian genetic algorithm (LGA). Initial position, orientation, and torsions of the ligand molecules were set randomly. Each docking experiment was derived from 10 different runs that were set to terminate after a maximum of 250000 energy evaluations. The population size was set to 150. During the search, a translational step of 0.2 Å, and quaternion and torsion steps of 5 were applied.

### Postdocking analysis and hierarchical clustering of compounds

The compounds are ranked by combining the pharmacological interactions and energy scored function of GEMDOCK. Hierarchical clustering method is based on the docked poses (i.e. protein-ligand interactions) and compound properties (i.e. atomic compositions). Atomic composition, which is similar to the amino acid composition of a protein sequence, is a new concept for measuring compound similarity. The output file was analyzed by treeview software.

### Statistical analysis

The data set obtained from the computational tools was correlated with RANi efficacy. Pearson's correlation coefficient and the significance of correlation were estimated by STATA statistical package (version 12.1). The results are provided in tables 3 and 4.

**Table 3:** Correlation analysis for RNAi activity and the measured parameters produced by iGEMDOCK.

	Total Energy	VDW	HBond	Elec	AverConPair	RL/FL
**Total Energy**	1.00					
**VDW**	0.833*	1.00				
**HBond energy**	0.4091	−0.08	1.00			
**Elec**	0.0188	−0.0593	−0.0198	1.00		
**AverConPair**	0.4799*	0.4168*	0.2372	0.2036	1.00	
**RL/FL**	−0.2255	−0.4153*	0.2279	0.1271	−0.4681*	1.00

*Pearson's correlation coefficient is calculated by STATA. Data showing significant correlation at 0.05 level are marked by asterisk.

**Table 4:** Correlation analysis for RNAi activity and the measured parameters produced by the docking server.

	Estimated free energy	Inhibition constant Ki	vDw + Hbond + desolvation energy	Electrostatic energy	Total intermolecular energy	Interaction surface	RL/FL
**Estimated free energy**	1.00						
**Inhibition constant Ki**	0.179	1.00					
**vDw + Hbond + desolvation energy**	0.7744*	−0.0341	1.00				
**Electrostatic energy**	−0.303	−0.1395	0.0237	1.00			
**Total intermolecular energy**	0.6443*	−0.1236	0.9642*	0.1918	1.00		
**Interaction surface**	0.1478	−0.5278*	−1.1483	−0.4163*	−0.2338	1.00	
**RL/FL**	−0.1681	−0.5756*	−0.1976	0.2236	−0.1478	0.5828*	1.00

*Pearson's correlation coefficient is calculated by STATA. Data showing significant correlation at 0.05 level are marked by asterisk.

### Other methods

Generation of protein surfaces, compounds electrostatic interactions were generated by Molegro Virtual Docker. Hydrogen bonding figures and binding site residues are generated by DS visualizer 3.1.

## Results and Discussion

### The rationale behind this study

During RNAi, siRNA binds and releases from its binding pocket of the PAZ domain of Ago proteins in a manner that allows proper coupling with the target mRNA and RNase activity. Unfortunately, little is known about the nature of such interactions. Although, stable or strong binding is expected to interfere with the release of siRNA from the PAZ domain, data investigated this process is lacking. Therefore, in this research, we tried to uncover the forces governing nucleotides recognition by the PAZ domain. We also correlated nucleotide-receptor specific aspects such as total surface of interaction, electrostatic forces, hydrogen bonding and interaction energy with previously characterized RNAi data.

### Docking results

**Figure 2:**
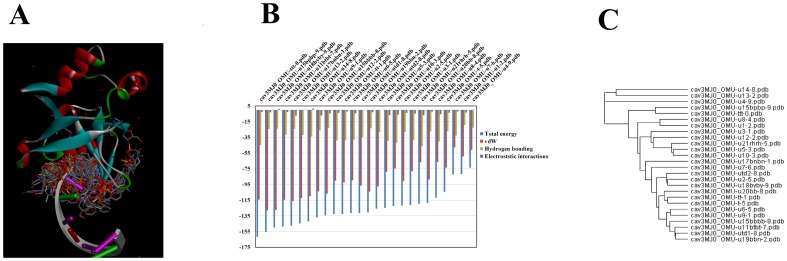
Docked poses of compounds with PAZ domain. Fig. 2A is a representative diagram showing the docked poses in the binding site of PAZ domain. From the docked poses, the best pose with the lowest energy was used to align with other compounds in the binding site of PAZ domain. The docking profiles of compounds ordered by total interaction energy are shown in Fig. 2B. The numerical values of data can be viewed in Table 1. The clustering of compounds is shown in Fig. 2C. The dendrogram is generated by iGEMDOCK post analysis tools and viewed by tree view software.

A representative figure of the best docked poses of compounds is shown in Fig. 2A. Before docking experiments, in either iGEMDOCK or the docking server the docking site was estimated and docking carried out against a predefined site that include residues within 10 Å from the center of the binding cavity. This was done to allow for possible interactions of compounds composed of dimers or trimers of nucleotides or nucleotide analogues. Furthermore, predefining the active site is helpful to get the best fit and most favorable conformation of compounds within the binding cavity [36].

Table 1 shows the results of iGEMDOCK docking runs. The compounds are arranged as given in Fig. 1. The last column indicates the ability of siRNAs to suppress gene expression, as determined by a dual-luciferase reporter assay with *Renilla* and firefly luciferase genes as reporter genes [22], [28]–[32]. All measured data are at 1 nM of siRNA concentration. The signal of *Renilla* luciferase was normalized to that of firefly luciferase. The tabulated parameters include total energy, vdW interactions, hydrogen bonding, average conpair and electrostatic interactions. Compounds ordered by their binding energy are further clarified in Fig. 2 B. Close inspection of this figure reveals that compounds showing the lowest total energy of interaction are usually dimers or timers (compounds ttt, u15bpbp, u18byby, u11btbt, u17bnbn). In contrast, compounds u4, u1 and u7 (which are docked as monomers) showed the highest total energy of interaction with the PAZ domain.

Similarly, output data obtained from the docking server are given in Table 2. Output data included free energy, inhibition constant, van der Waals interactions, hydrogen bonding and desolvation energy, electrostatic energy and interaction surface. Notably, the Ki value was not measurable for compounds u12, u13 and u14. These compounds are conjugated with a large hydrophobic substitution. Such bulky groups made it difficult to obtain accurate Ki values. Interestingly, these compounds are accompanied by relatively high RL/FL values of 0.6, 0.65 and 1.1, respectively.

### Clustering of siRNAs

**Figure 3:**
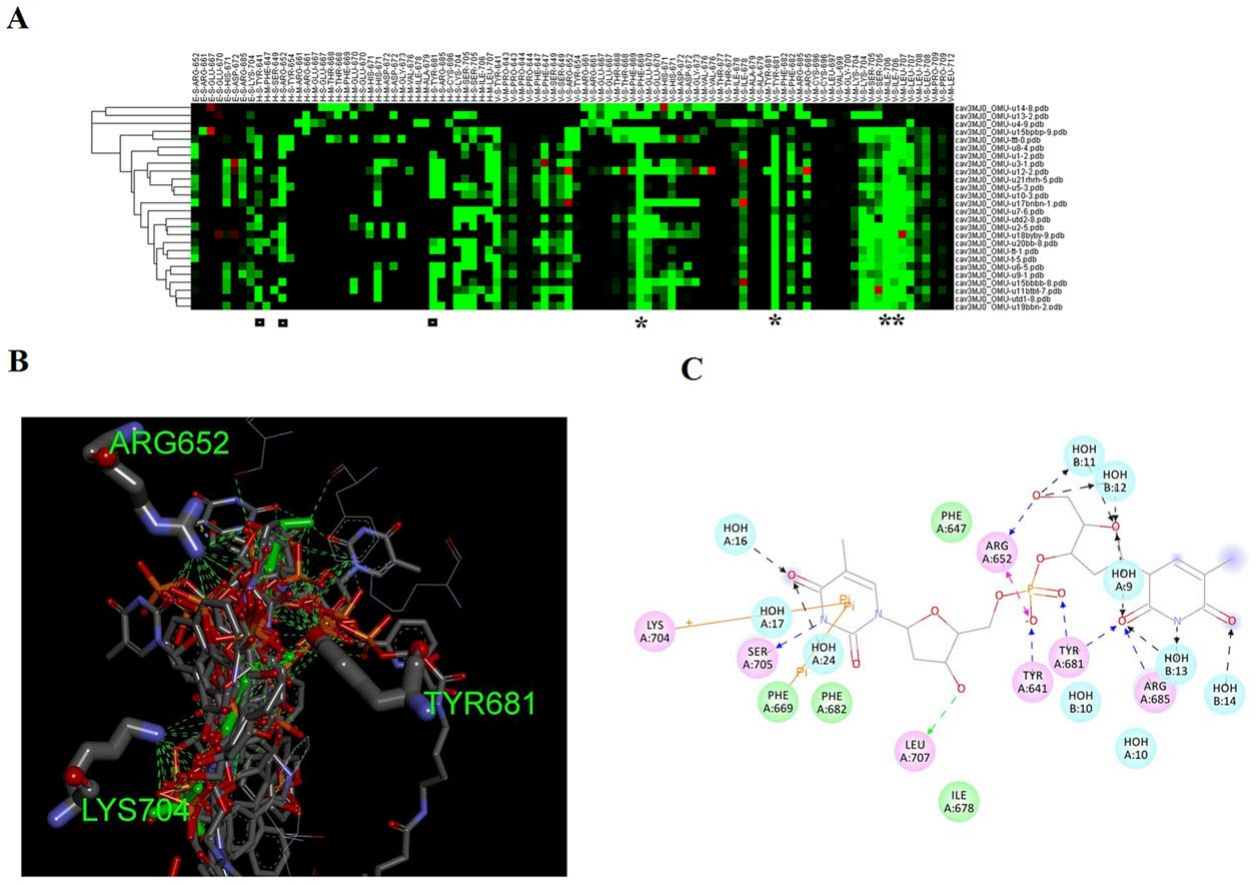
Interaction parameters of compounds with the PAZ domain. A Hierarchical clustering and the profile of interactions of various compounds with PAZ domain is represented in Fig 3A. The compounds are ranked according to pharmacological and energy-based scoring function. E, H and V indicate electrostatic, hydrogen bonding and vdW interactions, respectively. The vdW interactions are coloured in green when the energy is less than -4. The hydrogen bonding and electrostatic interactions are coloured in green if the energy is ≤ −4. M and S indicates main chain or side chain of interacting residues. The residues showing important hydrogen bonding interactions with docked compounds is given in Fig. 3B. Hydrogen bonds are represented as green dashed lines A two dimensional figure of binding of compounds with PAZ domain is shown in Fig. 3C. The ligand is compound tt, residues sharing with hydrogen bonding is shown in pink.

In conventional methods, the compounds are ranked according to energy scoring or affinity scoring system, in which the best candidates are performing the lowest energy or having uniquely high binding affinity. However, this is not always the rule, as the top of clusters might be compounds of similar structure and almost performing more or less binding patterns with the active site. By using iGEMDOCK post run analysis procedures, the compounds were clustered and scored on a generic evolutionary method. The clustering and building a dendrogram of the docked compounds is based on the interaction profiles including the atomic composition of the interacting partners (Fig. 2C). A dendrogram and interaction profiles of compounds is given in Fig. 3A. The figure summarizes the profile of interaction of each compound with residues of PAZ domain. Electrostatic, hydrogen bonding and van der Waals interactions of each compound with the main chain or side chain of each residue are assigned. The rule of each residue can be thoroughly assigned e.g. the most important van der Waals interactions is occurring with F669, Y681, I706 and L707 (indicated by asterisk), while Y641, R652, Y681 and K704 (indicated by rectangles) were important residues for hydrogen bonding (Fig. 3B). Figure 3C is a 2 dimensional plotting of the interaction of compound tt with residues of PAZ domain generated by ligand interaction scripts DS visualizer. The main coordinates of interactions agree with iGEMDOCK output (as mentioned above).

### Dissection of receptor-ligand binding forces

In dissecting the forces accompanying the recognition of siRNAs by the PAZ domain, docking output data obtained from 2 different sources (iGEMDOCK and docking server) were used. The validity of data were insured by several docking runs, repeated internal compounds as well as by using 2 different programs in obtaining the results. Interestingly, the data obtained from the two docking resources (iGEMDOCK and docking server) were in good agreement.

**Figure 4:**
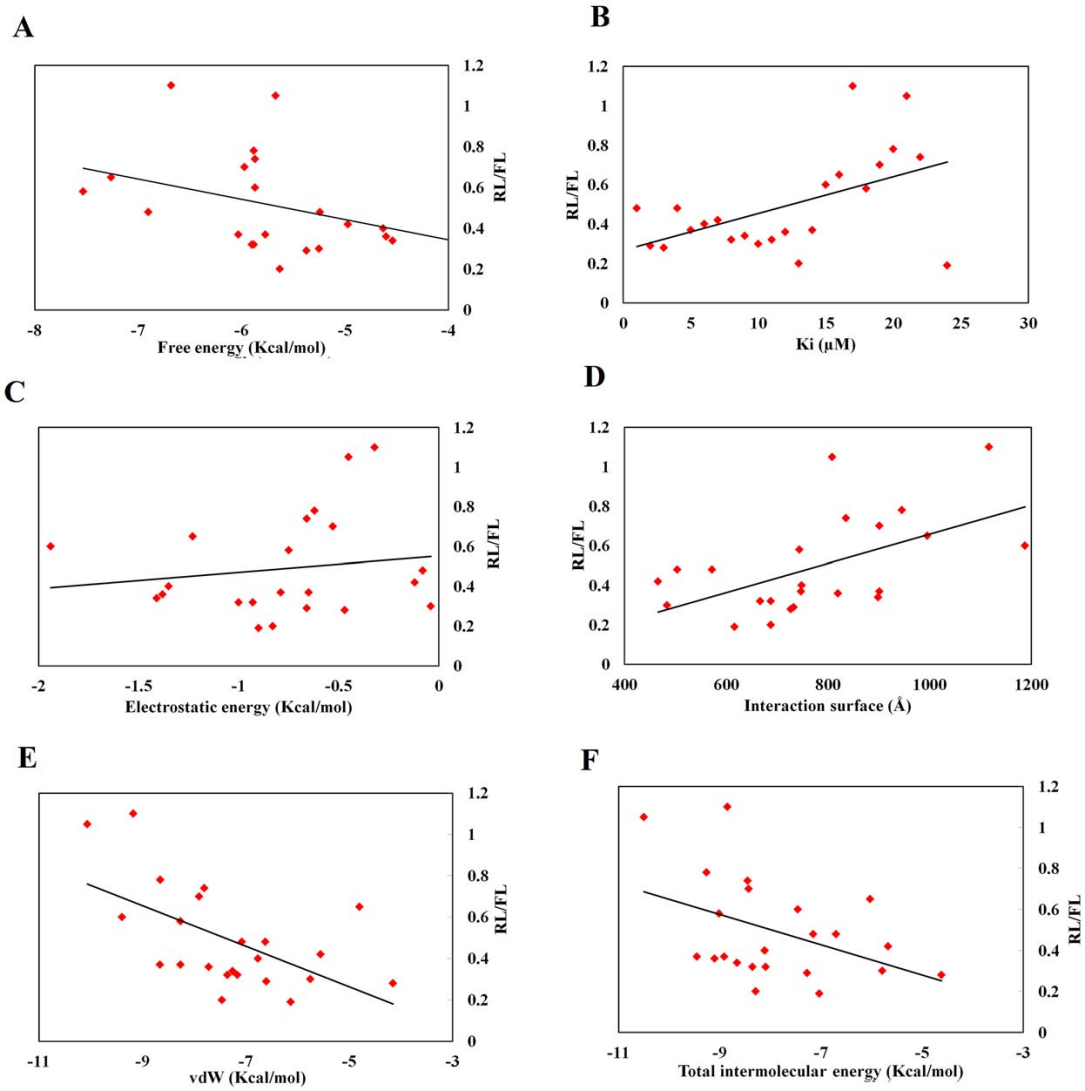
Dissection of the PAZ domain-ligands interaction forces (data is obtained from the docking server). Free energy (Kcal/mol), inhibition constant (µM), van der Waals interactions, hydrogen bonding and desolvation energy (Kcal/mol), electrostatic energy (Kcal/mol) and interaction surface were plotted against RL/FL (*Renilla* luciferace expression normalized by firefly luciferase data).

For correlating the previously obtained data on the suppression of gene expression with the results obtained from the docking server, the docking output data are plotted against the *Renilla* luciferase normalized by firefly luciferase data (RL/FL) in Fig. 4. The lower RL/FL indicates lower gene expression and potent RNAi activity. In contrast, higher RL/FL indicates lower potency of siRNA.

Correlation of free and total intermolecular energy: From Fig. 4A, we see that the lower values of free energy and total intermolecular energy (more negative) are accompanied by higher RL/FL ratio (lower RNAi efficiency). That is, lower free energy and total intermolecular energy promote better *in vivo* activity. We observe a considerable negative correlation between RNAi efficacy and the estimated free energy, albeit with a low Pearson's correlation coefficient (R = −0.168). The total and free energy are indicators of binding strength. Strong binding is associated with the release of free energy, which means lower binding strength is associated with better RNAi efficiency. Gu et al., suggested that PAZ domain acts as a handle to peel off the passenger strand in miRNA-like mismatch-containing duplexes [21], thus, suggesting a strong interaction of PAZ domain with siRNA. In this context, the PAZ domain was found to bind natural nucleotides with weak-to-moderate binding affinity [37]. Furthermore, the binding data from Ago1 and Ago2 indicates that PAZ is not a high-affinity nucleic acids-binding module [37].

Inhibitory constant (Ki): The compounds with a lower inhibitory constant are proposed to bind more strongly than those with higher Ki values. In Fig. 4B compounds with higher Ki values are associated with lower *in vivo* efficacy.

**Figure 5:**
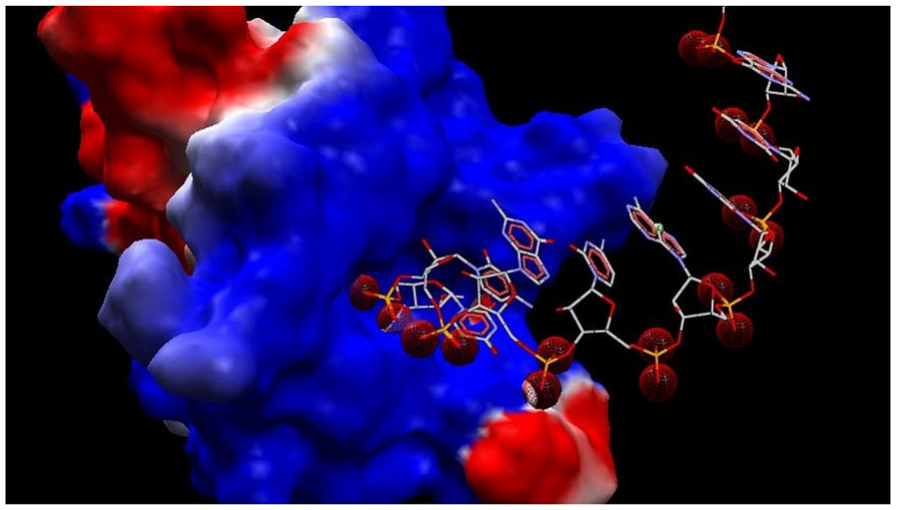
Electrostatic interactions of siRNA with PAZ domain. The phosphate groups of siRNA are marked by red colour opposing the surface of PAZ domain in blue.

Electrostatic interactions: From Table 1 electrostatic interactions are not a major contributor to the binding of compounds with the PAZ domain. Hydrogen bonding and van der Waals interactions are the major contributors to compounds recognition by the PAZ domain. Phosphate groups of natural nucleotides are the main source of electrostatic interactions with the cavity of the PAZ domain (Fig. 5). We found a moderate correlation between RNAi efficacy and electrostatic interactions (R = 0.22). Thus, we predict that modifying phosphate groups will not lead to major changes in compounds binding. In addition, their modification to other groups would be recommended if nuclease or phosphatase resistance is an obstacle to a compound's RNAi activity. Furthermore, electrostatic interactions showed little changes among compounds.

Total interaction surface: the interaction surface reflects the size of compounds and the area of the interaction surface with receptors. A higher area of interaction surface is associated with lower RNAi. RNAi efficacy was moderately-to-highly correlated with the interaction surface of the PAZ domain (R = 0.58, statistically significant at 0.05 level, Table 4). In agreement with the previously described work, this result suggests that smaller compounds are preferable for RNAi [10]. van der Waals and hydrogen bonding: these interactions contribute to the free energy of receptor-ligands binding, furthermore they stabilizes the binding process via association between electrically charged components of ligand-receptor complex. Higher RNAi was associated with lower van der Waals and hydrogen bonding interaction (Fig. 4E). RNAi efficacy was negatively correlated with the van der Waals interactions with the PAZ domain (R = −0.42, statistically significant at 0.05 level, Table 3).

**Figure 6:**
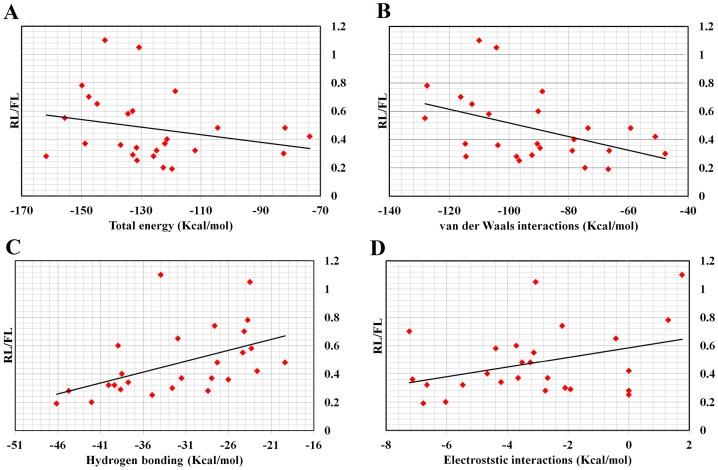
Dissection of PAZ domain-ligands interaction forces (data is obtained from iDEMDOCK software). The output data included total energy (Kcal/mol), van der Waals interactions (Kcal/mol), hydrogen bonding (Kcal/mol), electrostatic interactions (Kcal/mol) and average conpair plotted against RL/FL) and plotted against *Renilla* luciferace expression normalized by firefly luciferase data).

Results from iGEMDOCK are shown in Fig. 6. In a rough analysis of the figure, we concluded that higher RNAi activity is associated with lower values (more negative) of hydrogen bonding and electrostatic interactions and with higher values of intermolecular energy and van der Waals interactions. Within the measured parameters, the interaction surface, van der Waals interactions and inhibition constant showed statistically a significant correlation with the RNAi efficacy.

## Conclusions

In our investigation of the forces governing the recognition of siRNA by the PAZ domain and their *in vivo* association, we found that weaker binding is correlated with higher RNAi. Bulky modification of nucleotide favored low RNAi efficacy. This may be due to an unfavorable steric environment at the binding cavity of the PAZ domain. Through docking studies, we saw that the parameter of low total surface of interaction is associated with higher RNAi efficacy. A higher hydrogen bonding interaction was also associated with higher RNAi. Stronger hydrogen bonding is well known to be associated with a stronger binding interaction, however, based on other binding parameters, weak binding is still associated with better RNAi. Lower total intermolecular energy and free energy of interaction are associated with higher RNAi efficacy. Free energy and total intermolecular energy are more representative of binding strength since they represent the sum of forces involved in the intermolecular recognition. Thus, higher RNAi is associated with a weak binding process and is characterized by lower free energy of interaction, lower intermolecular energy, higher values of hydrogen bonding and lower Ki values. Based on our docking data, electrostatic energy is a minor contributor to the overall interaction energy, so replacing the phosphate group linking the nucleotides will have little contribution to the binding energy. In addition, such modifications would increase the resistance of the resulting compounds to hydrolysis by phosphatases. Findings from the present study should help guide the future design of modified siRNA analogues.
